# A Frequency Modulation-Based Taxel Array: A Bio-Inspired Architecture for Large-Scale Artificial Skin

**DOI:** 10.3390/s21155112

**Published:** 2021-07-28

**Authors:** Tareq Assaf

**Affiliations:** Department of Electronic and Electrical Engineering, University of Bath, Claverton Down, Bath BA2 7AY, UK; t.assaf@bath.ac.uk

**Keywords:** artificial skin, modular robotics, tactile sensing

## Abstract

This work introduces an array prototype based on a Frequency Modulation (FM) encoding architecture to transfer multiple sensor signals on a single wire. The use case presented adopts Hall-effect sensors as an example to represent a much larger range of sensor types (e.g., proximity and temperature). This work aims to contribute to large area artificial skin systems which are a key element to enhance robotic platforms. Artificial skin will allow robotic platforms to have spatial awareness which will make interaction with objects and users safe. The FM-based architecture has been developed to address limitations in large-scale artificial skin scalability. Scalability issues include power requirements; number of wires needed; as well as frequency, density, and sensitivity bottlenecks. In this work, eight sensor signals are simultaneously acquired, transferred on a single wire and decoded in real-time. The overall taxel array current consumption is 36 mA. The work experimentally validates and demonstrates that different input signals can be effectively transferred using this approach minimizing wiring and power consumption of the taxel array. Four different tests using single as well as multiple stimuli are presented. Observations on performances, noise, and taxel array behaviour are reported. The results show that the taxel array is reliable and effective in detecting the applied stimuli.

## 1. Introduction

The importance and transformative impact that spatial awareness in robotic platforms would have been well known since the concept of human like robots came to be. This critical need is recognized and well described in a work of two decades ago [1]. Lumelshy et al. acknowledges the fundamental need for robots to possess bodies covered with a sensitive layer as a means to be more efficient, spatially aware, and allow safe interaction with inanimate objects and people. The only solution envisaged, at the time, was to constrain the robot in a structured environment.

This design paradigm is still valid today for the majority of robotic and artificial platforms. With the exclusion of small robots deemed low-risk of causing harm due to low mass or speed, other platforms require specific training and/or a controlled environment. Therefore, when robotic platforms are designed to act or interact in the human space performance is severely limited resulting in reduced capabilities to make the interactions safe or safer.

More recently, the emergence and growth of care robotics, advancements in prosthetics limbs and associated research [2,3] renewed attention on this topic due to the need for closing the distance between platforms and users. New materials and advancements in tactile systems contributed to the flourishing of new studies, areas of interests, and applications, for example, the rising need for robots to take over complex and risky [4,5] tasks in hazardous environments to safeguard human lives. Tasks in radioactive or corrosive environments require spatial awareness and sensory feedback to be performed effectively.

Artificial touch has various challenges which include the area covered, number of sensors, and processing power. Even in localized solutions such as with touch pads, compromises have to be made. Studies on localized fingertip and tactile pads are the most common focusing on improving and providing fine manipulation and/or dexterity to robotic hands. Many examples of commercialized products can be found, and new ones are continuously developed and investigated.

Within the artificial skin field, studies can either focus on sensory systems to be applied on human skin or a sensitive coverage to envelop robots and platforms. This work and reviewed literature focuses on the latter which can be further subdivided into two classes: one aims to provide interactive interfaces to enhance human engagement by using soft stuffed “animals” and platforms [6,7,8] for haptic, interaction, and social studies; the other, more relevant work, focuses on providing sensory coverage to platforms. The coverage architectures can be either mainly centralized or mainly decentralized. The first is ideal for a limited number of sensors; while it easily reaches connection limits, it allows for direct and easy access to the sensor values. The decentralized approach delegates some computation to distributed electronics and collects the data into digital buses, which increases the potential number of sensors but is limited by the bandwidth of the bus selected. Despite the approach used, the ideal goal for large-scale sensory solutions is to create a system that can be part of a robotic platform. If the platform is stationary, power and processing can be externally provided through, for example, a tether. However, to be part of an autonomous platform, a system needs to be able to exploit the local resources (battery power, on-board processing units) or additional resources need to be added. If the resources are available or it is possible to add them, the system is feasible. However, depending on the demand, this can limit the speed or battery life. In this context, artificial skin needs to keep power consumption and processing to a minimum, while allowing for a large number of sensors. These are key characteristics for allowing deployment on autonomous platforms enhancing spatial awareness. This is highly challenging, and each approach mentioned is designed around priorities and compromises. For these reasons, while examples of systems exist, their number is limited due to intrinsic constraints and limitations due to scalability, and applications on experimental platforms can be found within laboratory settings.

The work presented here has characteristics of both approaches. It lays at the intersection of various disciplines, including electronics, bio-inspiration, communication, and signal processing. Therefore, the background and context will be presented from different points of view. This work has two main contributions to studies of large-scale artificial skin: first, a real-life use case scenario with evaluation of low-frequency stimuli and real-time sampling and reconstruction, and second, the work showcases a completely new and different approach from current mainstream solutions and represents a unique design for internal robotic communication systems applied to sensory arrays.

The taxel, used in this work, exploits the Frequency Modulation (FM) technique to transfer the sensor signals (constituting the taxel array) over a single wire with extremely low power demand. The architecture working principle is described in the materials and methods section. A collection of alternative architectures and solutions are presented in the background section to provide a reference point.

The taxel array unit used in this work aims to showcase a single use case scenario and evaluate the performance of both the taxel array and architecture. In this example, eight Hall-effect sensors are used. One of the key features of the architecture is that any other sensor that outputs a voltage signal could alternatively be used. Other types of sensors can be made compatible after appropriate signal conditioning to generate proportional voltage variations.

The FM technique is not new, and it has been used for decades in radio and wireless communication background. However, the architecture shows intriguing and unique characteristics by repurposing this wireless methodology into a wired unidirectional robotic internal sensory bus/network. The taxel system has a layered structure inspired by natural skin. These two characteristics are inspired by natural tactile sensory systems.

**Figure 1:**
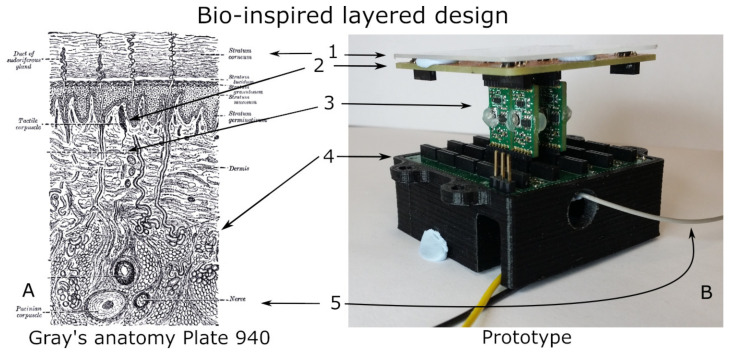
The image presents an illustration of the skin cross section (**A**) and side view of the prototype (**B**) layered structure. (**A**) Henry Vandyke Carter—Plate 940, Gray’s Anatomy: Descriptive and Surgical book [9] (Wikimedia image—public domain—some labels have been removed for clarity). (**1**) Outer skin interface; (**2**) sensors/nerve endings; (**3**) axons and encoding structures; (**4**) substrate/mixing signal and (**5**) nerves/main wire.

As visible in Figure 1, both the skin and the prototype used are built in distinct layers. From top to bottom, there is a protective layer, sensors, encoding and communication, a mixer, and transmission to a central system. The design takes its inspiration from the neuromorphic approach of biological tactile systems, which integrates sensing, filtering, and preprocessing. In the design, this translates to sensing, filtering, and encoding, while the preprocessing is a desirable feature, it has not been implemented due to the higher priority of power and footprint reduction.

The paper is structured as follows. Section 1.1 presents a brief overview of various key state-of-the-art elements and topics relevant to this work. Section 2 presents the materials and methods used in this work. Section 3 presents the results and observations. The performance of the taxel array is discussed in Section 4. Last, Section 5 summarizes the conclusions of the study.

### 1.1. Background

Artificial skin-like systems have seen a growing interest in different fields (e.g., wearable, thin, soft artificial skin for human-oriented applications) thanks to novel materials and techniques. However, large-area skin-like systems for robot spatial awareness are fewer in number and mainly limited by the issues of scalability that make deployment and use very challenging.

According to the literature, unresolved technical open issues [10] remain related to *scalability* and *deployment*. *Scalability* has a series of related issues which include power, costs, signal reconstruction, design, and maintenance complexity. *Deployment* includes difficulties in creating and integrating a unique design of conformable and compliant coverage for different platforms.

The different subsections aim to contextualize the three main components and concepts that enable the presented taxel action: the FM technique, artificial tactile systems, and proposed architectures for large-scale skin deployment.

#### 1.1.1. FM Modulation Technique

A 2016 work shows a FM modulation–demodulation approach used to acquire and transfer an ECG signal in real-time that could be easily connected and decoded using an audio PC card [11]. This work [11] uses relatively low frequencies (20 Hz to 20 kHz) and transfers/decodes a single signal.

FM radio (8 MHz to 108 MHz), on the other hand, is capable of transmitting high number of signals at high frequencies, over great distances. The FM radio technique, introduced many decades ago, is therefore reliable and well known, for example, it has good noise rejection. The radio receivers have been designed and perfected to single out a “station” or signal.

The architecture and transmission strategy used in this work falls between these two extremes. The main differences include the modulation of a large number of signals as well as the demodulation of all signals at once. Two characteristics add higher complexity with respect to the other two applications. The single-wire transmission approach reduces power demand and looming within the system. Last, the frequency range used is medium–low (8 kHz to 1 MHz), which facilitates the development and design of electronics while avoiding some of the constraints and issues caused by operation at radio frequencies. The system also benefits from the deep understanding of potential interference and noise related to FM transmission for which techniques to avoid or fix them have been developed and proved valid in radio. Literature and resources on channel intermodulation interference are not limited to FM radio techniques but also include wireless and frequency modulation communication in general. These can be affected by noise caused by a variety of sources including components, number of transmitters, or harmonics [12,13,14,15]. Due to the nature of the application and signals this effect has limited impact on the presented work.

#### 1.1.2. Artificial Touch

Tactile systems are many and exhaustive reviews can be found in the literature. An example is [16] in which Lucarotti et al. reviews synthetic and bio-artificial tactile sensory systems. In other examples [17,18,19,20,21] the topic is approached from different points of view. All these works showcase new technologies and sensory systems based on rigid, semi-rigid, and soft materials aimed to emulate natural systems.

In most cases of tactile systems, regardless of the sensor typology used, the sensors are strictly interlinked with the system architecture. This is needed because different sensors often require specific hardware to be properly acquired and most importantly sent to the processing unit. A recent example is Skin-On interfaces [22]. Teyssier et al. present a self contained soft touch patch pad and envisions primarily the use in common interactive devices such as PC, smartphones, and smartwatches but also suggests the use to interact with robots and other objects.

#### 1.1.3. Large Scale Tactile Architectures

Existing artificial skin for robotic platforms is the last element required to fully contextualize this work. Two types of architectures and proposed solutions to skin scalability are considered. The first collates solutions that minimize the distributed electronics by delegating the computationally intensive tasks of discriminating and reconstructing the data to neural networks and learning algorithms. The second collates those solutions in which the distributed electronics have higher functions and complexity. These are used to acquire, process, and serialize on digital buses the sensory data to a central control unit.

Although both types are very different from the underpinning principles used, the first will be briefly outlined for completeness, and the second will be used as a point of reference because it is closer in terms of distributed architecture layout to the one proposed.

- Neural network-based systems
- Distributed electronics and digital bus

**Neural network-based** systems aim to reduce power and looming via simplifying electronics as an artificial version of mechanoreceptors. The downside is that the signal is highly complex and the processing requires a higher level centralized approach normally based on neural network-based algorithms [23,24,25]. This approach, although valid, might create a situation where transparency or modifications might be complex. Within these systems the signals are acquired and mixed in a single conductor. The signals can be action potentials-like signals as in [24,25] or multiple resistive sources as in [23]. The resulting composite signal requires neural networks to decouple the various sources and identify specific patterns. Without the level of abstraction and computation of a neural network the original signals would be difficult, if not impossible, to retrieve.

**Distributed electronics and digital bus** are considered representative of the mainstream approach and three architectures are selected to present alternative solutions as a comparison: iCub artificial skin, Cut-and-paste tactile sensors, and Hex-o-skin.

In the iCub artificial skin [26,27,28,29,30], the skin comprises triangular units, with each unit housing 12 capacitive sensors. An ideal 192 taxels can be connected using the CAN bus protocol and subnetworks can be used to increase the number of sensors. The units have been used on the iCub platform to envelop arms, legs, and torso.

In the Cut-and-paste tactile sensors [31] the skin features decentralized microcontrollers. Scaling power consumption for this architecture is difficult and needs to be managed applying time-sharing control over the sensor elements (e.g., 50 A to supply 1000 tactile elements). The skin related to this architecture is sensitive to both contact and pressure through LED light variations. This architecture is built on a flexible PCB and it can be cut and tailored to fit a specific layout.

In the Hex-o-skin [32,33,34], single hexagonal cells have a dedicated microcontroller and several sensors (7) including temperature, acceleration and proximity. In this case, the power consumption can be high and power management strategies to mitigate the energy used by the LEDs is needed.

All these architectures use digital communication systems and buses. Microcontrollers are used as an intermediary between sensors and the processing unit to encode and transmit the information digitally.

In contrast, in this work a low-power integrated circuit (IC) is used to achieve analogue FM encoding on a single wire. The digitalization is deferred to the central processing unit. Table 1 presents a brief summary of the key criteria compared with the transmission strategy used in this work.

These solutions have different advantages and disadvantages, and the choice of using one solution or another is strictly related to the task and desired specifications. For example, CAN bus solutions or microcontroller solutions, in general, have advantages during development and configuration, e.g., many changes can be made via soft- or firmware level. Buses also allow for bidirectional communication which is lost in FM. However, apart for debug/development and configuration purposes the loss of downstream communication is both consistent with natural systems and largely compensated by reduced power requirements. The FM encoding can be configured but physical changes (e.g., resistors) are required making it less “flexible”, however once the skin is tailored to a robot it should not need changes. Last, the CAN bus, taken as example, has physical limitation by design which limits the number of sensors and/or update rates. Other faster buses can be used but the electronics to handle such speeds requires higher power and therefore a trade-off needs to be reached. The FM architecture has limits, but the bands can be simply changed and adapted to the specific task. For these reasons, the FM architecture has potential advantages when a large number or variety of sensors need to be used.

Compared to the two aforementioned classes of approaches, the proposed solution sits in the middle bridging the hard choices between IC based distributed systems to collect and stream the data, and high complex decoding learning-based strategies which hold great relevance but sometimes can lead to reduced transparency, constraints and complexity if changes are needed.

The proposed solution sees a reduced complexity of the distributed system with competitive reduction of power demand by maintaining a relatively easy access to the information encoded in the FM signal which can be retried numerically without the need of neural network and learning providing in effect a direct parallel measurement of the signals. Of course higher level computation, e.g., neural networks can be applied to these signals if needed afterwards for high level control purposes but the data are also available to adopt other control strategies.

## 2. Materials and Methods

**Figure 2:**
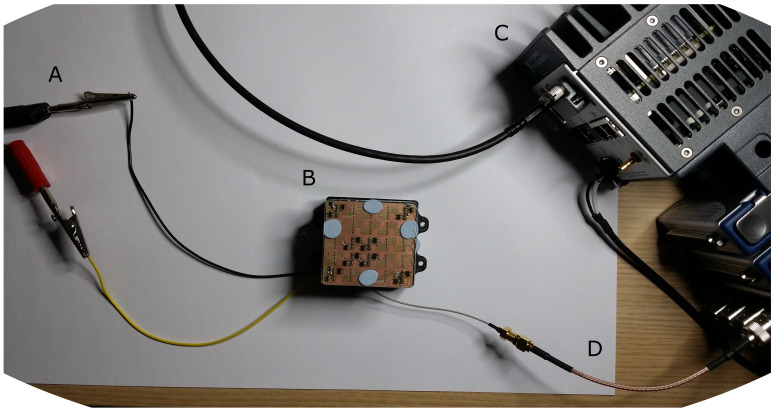
Experimental Setup. (**A**) Power from external supply 5 V. (**B**) Sensors and encoding architecture. (**C**) Compact Rio 9045 used to acquire and demodulate the mixed FM signal. (**D**) Single wire connection to transfer the 8 sensors signals.

The setup is visible in Figure 2 and comprises 4 elements: the supply (5V) (Figure 2A), the prototype unit (Figure 2B), the acquisition and processing system based on a Compact Rio (CRio) 9045 from National Instruments (NI) (Figure 2C), and the single wire that connects the taxel array to a C Series digitalizer module NI-9775 (Figure 2D).

The experiments have been sampled at 250 kHz, but NI-9775 can sample up to 20 Mega samples per second (20 Msps). At the moment, the processing is executed on the CRio real-time embedded system. The system has an FPGA on board which is not currently used. This choice, although limiting the number of sensors that can be used, is favored to allow for higher flexibility and transparency in this phase of the investigation and assessment.

The prototype (Figure 2B) is designed for up to 5 × 5 encoding units in a 5 × 5 cm. In this series of experiments 4 boards are used. Each board can encode two signals for a total of 8 signals. The boards and 8 sensors use 36 mA (3–4 mA for the sensors and 1–2 mA for each encoding board).

**Figure 3:**
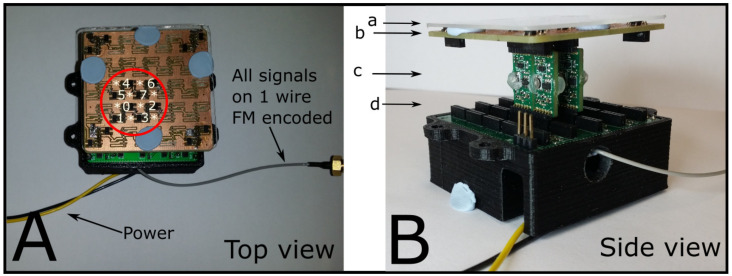
(**A**) The figure shows 8 Hall-effect sensors (circled and numbered) used in this test within a 10 × 10 matrix test rig. Channels are numbered from 0 to 7 and have carrier frequency of 8, 11, 14, 17, 20, 23, 26, 29 kHz respectively. * represents virtual sensors used to complete the 4 × 4 taxel elements calculated as intensity average of neighbors after decoding. (**B**) side view. (**a**–**d**) Protection layer, sensor and connection board, encoding boards, and mixing board, respectively.

In Figure 3A the active sensors are shown in the circle and numbered from 0 to 7. The sensors are also identified by their channel also indexed 0 to 7 (channel carrier frequencies are 8, 11, 14, 17, 20, 23, 26, and 29 kHz, respectively). A transparent polycarbonate (Figure 3B-a) screen simulates the skin protective layers ensuring that the magnets used to trigger the Hall-effect sensor response could move freely without damaging the sensors and maintain the same distance which allows for consistent and repeatable stimuli. Hall-effect sensors are selected because taxels using this technology exist [35]. Although the taxel in this work is highly simplified it also allows both the testing of the architecture as well as testing more realistic scenarios in sensing magnets at varying distance through the observation of the signal from neighboring sensors. Evidence is presented in the result section, Section 3.4 (Magnetic gradient and sensors response). Each sensor is connected to an FM encoder board (Figure 3B-c) with its own carrier frequency. The carrier frequencies are spaced at 3 kHz intervals. Each channel has a bandwidth of 3 kHz comprised of ±1 kHz channel deviation +500 Hz spacing each side.

**Figure 4:**
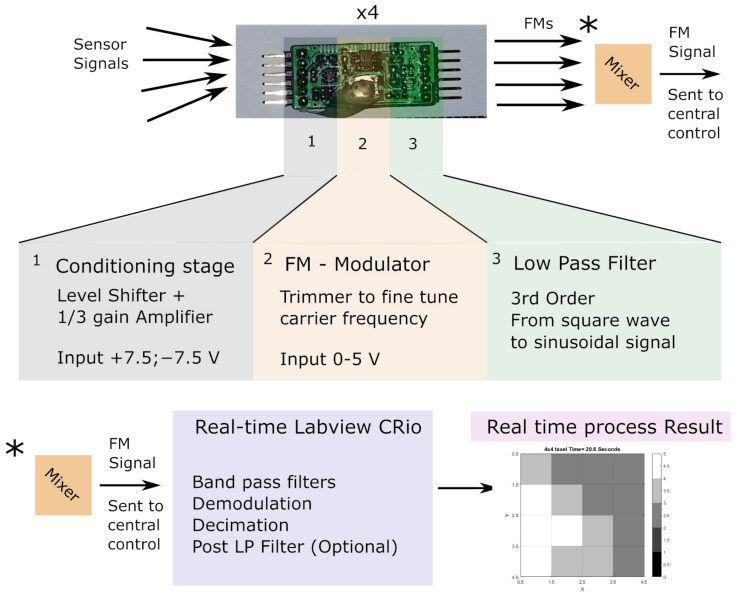
Diagram of the encoding board and the demodulation process. From top left to right encoding and bottom left to right decoding and taxel output. The asterisk (*) indicates the equivalent point in the modulation (**top**) and demodulation (**bottom**) processes.

Any sensor that outputs (conditioned or native) a voltage signal can be used with this architecture. The different layers are visible in Figure 3B. Figure 3B-b is a connection board that allows the interface between the sensors and the encoding units, Figure 3B-c is the encoding unit, and Figure 3B-d is a second connection board that mixes all the channels into a single FM mixed stream and sends it via a single coaxial wire to the acquisition and processing unit. The mixing board also hosts a voltage reference source at 2.5 V for the level shift conditioning stage (see Figure 4).

The boards and prototype have been configured for a signal of ±7.5 V to enable a wide range of tests. To achieve this the conditioning stage has a 2.5 V level shifter with a 1/3 gain amplifier configuration that scales the inputs voltages to the IC range (0–5 V). The boards are not configured or optimized for the specific sensors. For these Hall-effect sensors conditioning would not be needed (the output ranges from around 1 to 4 V). This does not affect the end result; it would just change the ratio between signal and frequency deviation. The prototype is configured for a more general multipurpose application. The Hall-effect sensors are tested in these conditions to prove that the system works regardless, highlighting its flexibility and robustness.

**Figure 5:**
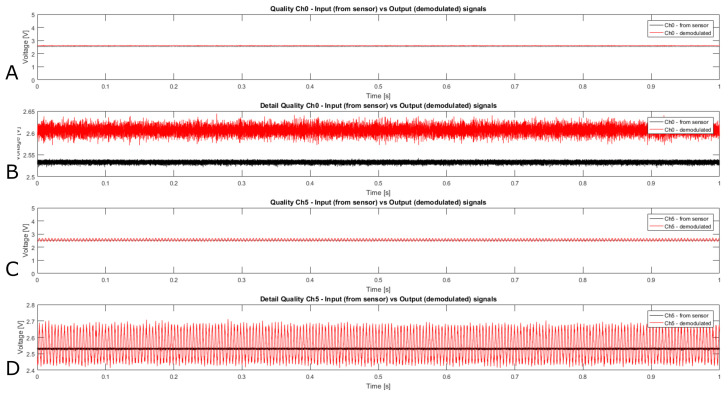
(**A**) Channel 0 Input signal (measured directly from sensor) and output reconstruction after demodulation without any adjustment. (**B**) Plot detail of A. (**C**) Channel 5 Input signal (measured directly from sensor) and output reconstruction after demodulation without any adjustment. (**D**) Plot detail of C.

Figure 5 presents two examples of input signal and output reconstruction. The two channels selected are Ch0 and Ch5. Ch0 is taken as an example for all the channels except Ch5. Ch5 would have similar output but it is affected by periodic noise which will be discussed in the following. The offset is due to the slight difference between the carrier frequency (tuned using the trimmer on the board) and the frequency used within the demodulation stages. Table 2 reports the signal amplitude for both channels. The amplitude of the noise directly measured from the sensor (black lines) is ~23 mV and the one from the demodulated signal is ~70 mV for those behaving like Ch0, taken as an ideal example. These amplitude variations are quite small 0.8% or 2.3% over the 3 V sensor range. The comparison of Ch0 input and reconstructed signal show that the quality of the signal is not particularly affected by the FM modulation/demodulation.

In Figure 4 a block diagram of the system is presented. Each encoding board can be divided into three stages: conditioning, encoding, and filtering. The conditioning stage can be tuned to accommodate a variety of sensors. The IC used for FM encoding produces a modulated square-wave which could be used but it would generate too much interference and undesired harmonics. Therefore, a 3rd-order passive filter is used to smooth the output FM signal and obtain a FM sinusoidal output.

After mixing, the FM signal is sent to the acquisition system (CRio Real-time core) which uses a real-time engine to perform software-based filtering and demodulation of all 8 sensors simultaneously.

The bandpass filter extracts each channel band. The singled FM waveforms are processed by NI labview signal processing blocks (e.g., downconvert) which extracts the quadrature (I/Q) complex components. These blocks shift the singled FM centered to the specific channel to the base band. The complex signal is then fed into Equation (1) [36] which can extract the modulating signal (input signal). A simple decimation is used to reduce the output sample rate and memory usage:

(1) $$f_{sig}=\frac{\frac{\delta }{\delta t}\left(Im_{fm}\right)\cdot Re_{fm}-\frac{\delta }{\delta t}\left(Re_{fm}\right)\cdot Im_{fm}}{{Re_{fm}}^{2}+{Im_{fm}}^{2}}\cdot SF$$

where $f_{sig}$ is the demodulated signal and SF is a constant scaling factor. In this work, −7.5 was used (to remap the ±7.5 V configured range). The negative sign is used to compensate for VCO Inverted logic. $Re_{fm}$ and $Im_{fm}$ are the real and imaginary part of the FM I/Q signal, respectively.

The taxels’ signals are then converted into a 2D 4 × 4 gradient map. The real-time calculated values are also logged for further analysis and replay of the experiment. The data log has been used to playback the taxel array activity. In order to generate a 2D 4 × 4 map of the taxel array a further 8 locations have been generated in real-time as the average of neighboring sensor signals (Figure 3A * locations). The 2D reconstruction has been adopted in this work because it is easier to visualize. However replay of the data using a 3D surface is also possible.

The magnet was moved manually to simulate excitation events to evaluate the taxel array and architecture response.

## 3. Results

This section presents the results and observations of the taxel array tests.

Five groups of data are presented using data acquired in four tests. The following subsections present:

- Taxel reconstruction—the output of the taxel array is overlaid with video recorded during the experiment.
- Single sensor evaluation—the complete output of two experiments in which each signal or channel is presented.
- Ch5 (signal at 23 kHz) noise—data collection for which a post filter on channel 5 is turned ON and OFF.
- Magnetic gradient and sensors response—correlation between signal intensity and sensor distance from magnet.
- FFT mixed FM signal analysis—two mixed FM signals, from two experiments, for which frequency components have been analyzed using a Fast Fourier Transform (FFT). The mixed FM spectrum is analyzed and the various frequency components clearly visible.

### 3.1. Taxel Reconstruction

**Figure 6:**
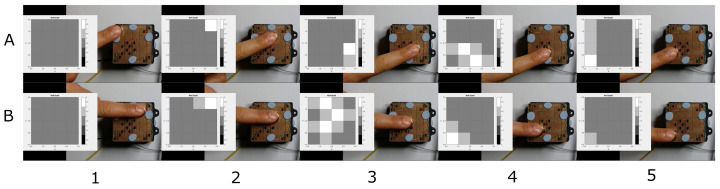
(**A**) Sequence of magnet circular movement (outer edge circle clockwise). (**B**) Sequence of diagonal movement from top right to bottom left corner.

Figure 6 shows two snapshot sequences. The two movement patterns, (A) circular and (B) diagonal, are correctly reproduced by the taxel array. A video was captured during the experiments and manually synchronized. In the figure, the taxel array is reconstructed, using MATLAB, from the data logged during the experiment.

**Figure 7:**
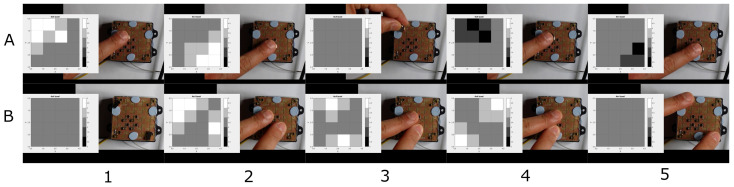
(**A**) Sequence of diagonal top left-bottom right then, change in magnet polarity and same diagonal path. (**B**) Sequence of the movement of two magnets.

Figure 7 shows a second series of acquisitions using (A) the same magnet as previously but alternating the polarity to show a pull/push output and (B) two smaller magnets to simulate multiple simultaneous contacts. As previously, the figure presents five snapshots of the experiments.

### 3.2. Single Sensor Evaluation

**Figure 8:**
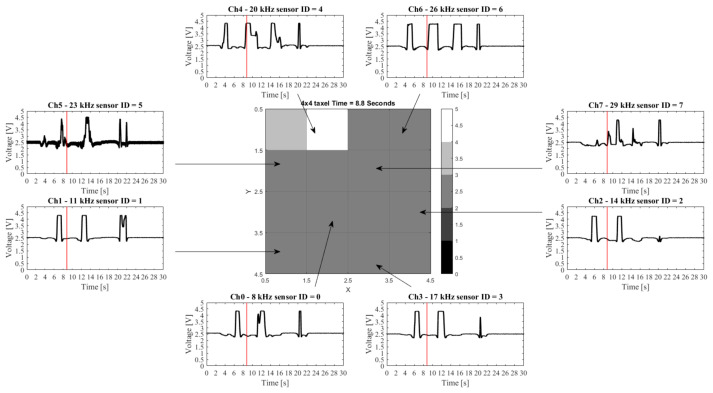
Example of data being transferred and demodulated. The 8 channel signals and corresponding associated squares are indicated. The taxel array image is taken at a time of 8.8 s. The line in each plot shows the individual sensor values at that point in time.

**Figure 9:**
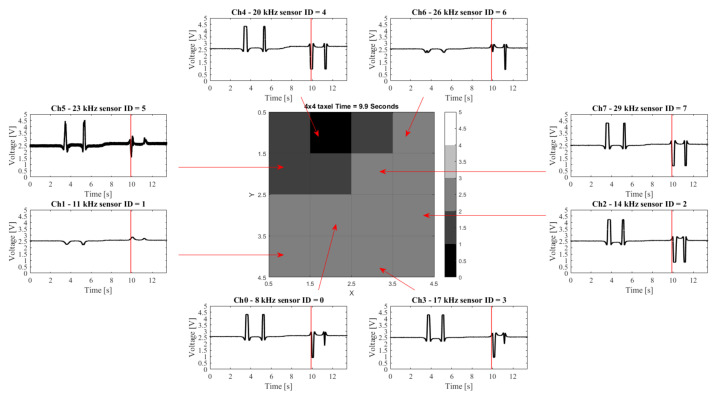
In this example positive and negative values are visible in the data. The 8 channel signals and corresponding associated squares are indicated. The taxel array image is taken at a time of 9.9 s. The line in each plot shows the individual sensor values at that point in time.

Figure 8 and Figure 9 show the data acquired in two experiments and the corresponding taxel array output at a specific time frame (in the examples 8.8 and 9.9 s are used, respectively). It is immediately visible that despite the noise on channel 5, the signal variations are clearly visible in all channels. The noise and repeatability of the measurements can be assessed.

### 3.3. Ch5 (Signal at 23 kHz) Noise

**Figure 10:**
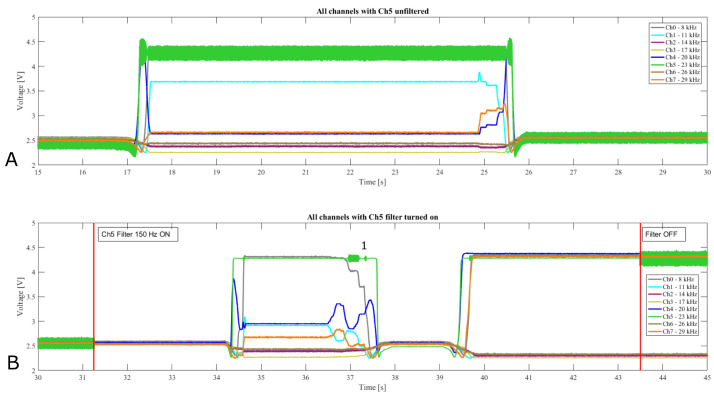
Figure shows a data acquisition from all 8 channels from 15 to 45 s split in two plots (**A**,**B**). (**A**) Post-filter on channel 5 is OFF. (**B**) Between seconds 31 and 32 the filter was switched ON and then OFF between seconds 43 and 44. This shows how the noise induced from channel interaction could be removed to clean Channel 5 which is affected.

Figure 10 presents the acquisition during a single experiment where a low pass filter was applied to the decoded channel 5 signal. Figure 10A highlights a section from 15 to 30 s without the filter and Figure 10B from 30 to 45 s where the filter was toggled ON and OFF. The filter effect on Channel 5 is clear and improves the output considerably. There are still small aberrations, as shown at the point Figure 10B-1 but the result is satisfactory. The noise is caused by harmonics induced by the lower frequency channels, which was expected. Encoding board refinements and optimizations of the output filter stage should reduce these effects eliminating the need for the post-filter; however, even in this early prototype stage the output is sufficiently clear to detect the stimuli changes.

### 3.4. Magnetic Gradient and Sensors Response

**Figure 11:**
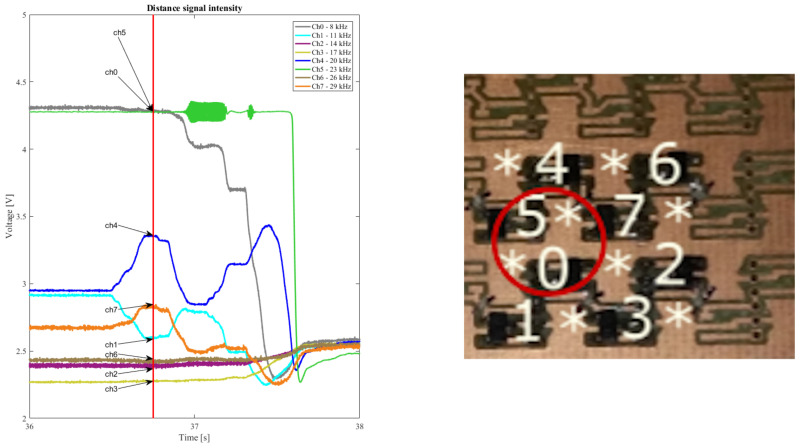
The image on the right shows the sensor layout. The plot shows a portion of the recorded signals during which the magnet is present and then removed. In the instant (vertical line) around second 37 the magnet is hovering over Ch5 and Ch0 (e.g., around the circled position) as shown by the maximum signal on these channels in the plot. The other neighboring sensors show decreasing signal correlated to the distance to the magnet and magnetic field intensity.

Another interesting observation is the capability of the system to correctly recognize magnetic gradient related to the distance of the magnet. Figure 11 presents a portion of Figure 10B and the sensors layout. Due to the sensor layout and simple use case scenario selected to assess the system performance, the magnet maintains a constant vertical distance between magnet and sensor, which saturates the sensor output of those directly underneath the magnet. However, the neighboring sensors show signals correlated to the distance and influence of the magnetic field.

### 3.5. FFT Mixed FM Signal Analysis

**Figure 12:**
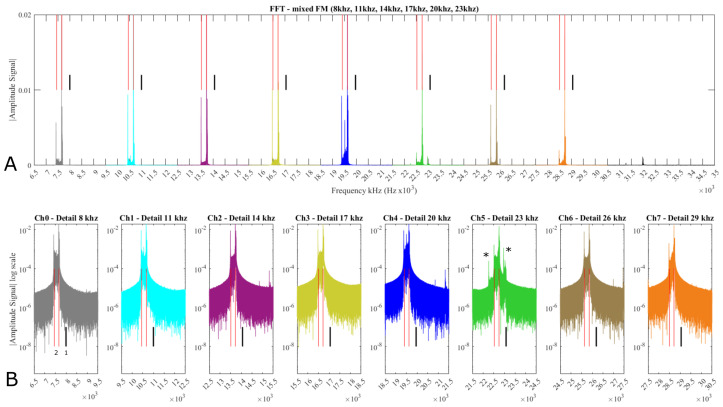
FFT analysis of the mixed FM signal. (**A**) represents the FM spectrum in normal scale. (**B**) in Log scale. The small vertical line (in black (1)) shows the carrier frequency position. The two thinner red lines (2) identify the peaks of the frequency deviations at −333 and −500 Hz from the carrier frequency that correspond to the sensor outputs 2.5 and 4 V, respectively.

**Figure 13:**
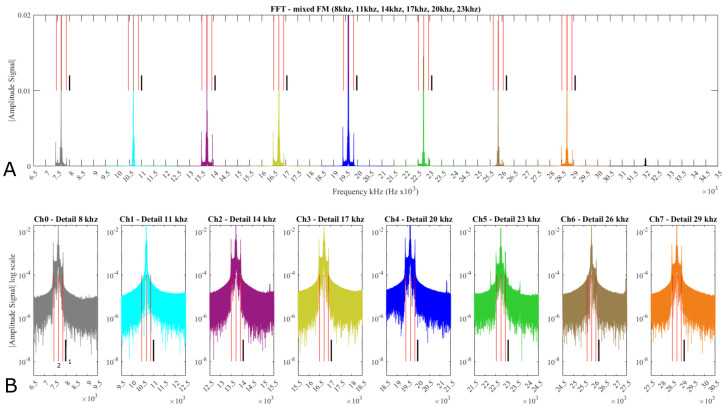
FFT analysis of the mixed FM signal. (**A**) presents the FM spectrum in normal scale. (**B**) in Log scale. The small vertical line (in black (1)) shows the carrier frequency position. The three thinner red lines (2) identify the peaks of the frequency deviations at −100, −333, and −500 Hz from the carrier frequency that correspond to the sensor outputs 1, 2.5, and 4 V, respectively.

Figure 12 and Figure 13 show FFT spectra for two different experiments. These plots show that the various frequencies are clearly visible and the center frequency (black line) represents 0 V signal (it is absent due to sensor working output ranges). In Figure 12, the predominant components at 2.5 V and ~4 V (which are the base signal and max positive Hall-effect values) lay as expected around −333 Hz and −500 Hz off the center frequency, respectively. In Figure 13, the predominant components are 2.5 V, ~4 V as before but a third peak at −100 Hz off the center frequency identifies the ~1 V output, resulting in a negative value when the magnet polarity is changed.

These values can be linearly calculated as 1000 Hz deviation/7.5 V * voltage measured, which was expected. The negative sign is due to the IC inverted logic (higher the voltage lower the frequency). This result, although expected, shows the repeatability among the 8 channels.

Figure 12B and Figure 13B show the FFT spectra of the mixed FM signal in a linear and log scale, respectively. Each channel, shown in Figure 12B, has been singled, in individual plots, to highlight the bands and show that in Ch5 there is evidence of a Ch0 third harmonic and other unwanted peaks (Figure 12B Ch5 (*)) which are causing the noise (~250–300 mV) on Ch5 as shown in Figure 5. These peaks are also present in Figure 13B but harder to spot due to the overlap with Ch5 valid frequencies.

Figure 13 presents another FFT spectrum for the experiment in which the magnet was used with both polarities. Similarly to Figure 12B the channels are singled out and another peak is visible representing the inverted polarity.

## 4. Discussion

This work showcases a use case application of a taxel based on FM encoding capable of providing real-time data acquisition. The FM architecture allows for compact communication to a central processing unit over a single wire.

One of the key points of reducing wire number in artificial skin structures is that for large numbers of sensors wire management (e.g., number, connections, mechanical fatigue, and space usage) becomes paramount. This applies to (i) the encoding units’ connection to the central control, which in the FM based architecture is one coaxial wire and current systems using digital protocols have 2 to 4 wires. Multiple subunits can be used and therefore the wiring of FM-based architectures scale at a slower pace; (ii) the connection of sensors to the encoding units and although linked to the overall design and task, minimization of footprint and connections are ideally the goal (e.g., sensor close or on the same board). Some solutions present instead several sensors connected to a single microcontroller via leads which is an acceptable solution that provides advantages such as reach and reduction of encoding elements. However, for large numbers these cannot scale as well due to encumbrance, power and costs. Scalability potential is therefore skewed towards FM in terms of power, wire reduction and reduced board footprint. Where the FM system falls short is that its benefits only become apparent in the context of a large number of sensors. Considering that this is the end goal of artificial skin (collecting information from many thousands of sensors), this was expected and accepted during design.

The five sets of results presented highlight the good quality of signal reconstruction, and the feasibility and robustness of the system.

The taxel array sequences (presented in Section 3.1) show how the experiments were performed. The reconstruction examples provided as support material relate to the sequence in Figure 6A,B. At the moment the 2D 4 × 4 taxel array is built using sensory data and a simple neighboring average. A larger array and more advanced techniques would enhance the resolution of detection.

Comparing all channels (Section 3.2), the data acquisition quality and effective simultaneous parallel processing is visible. In these plots, the signal to noise ratio of all channels can be appreciated and Ch5 is visibly affected by higher noise. This noise is expected and well documented in FM. For this application mitigations are not required, however, to prove the quality and effectiveness of the proposed system a post filter was used to clean the signal. The result (Section 3.3) of the filtered/unfiltered signal shows how a simple filter can improve the output. However, it is also evident that even without a post filter the profile and rate of change is clearly detectable. Initial observations of the signal noise ratio show that it is very low, with the exception of Ch5, but still sufficiently low to discriminate sensor activity. The same filter could be applied to all signals but it is not necessary. Possible sources contributing to noise on the reconstructed signals include (i) the analogue nature of the sensors which introduce noise and (ii) the computation noise/errors introduced during the FM demodulation. Other sources of noise can be excluded because, as previously mentioned, FM is inherently resistant to noise and removing Ch0 causes the noise on Ch5 to almost disappear.

The observation in Section 3.4 about the signal correlation between distance and Hall-effect sensor output highlights the sensitivity and encoding accuracy of the system. The current X–Y sensitivity demonstrated would extend to Z-axis. Whilst no further processing has been performed, it is evident that a wider range of information is present within the signals including direction of movement and speed.

Finally, the inspection (Section 3.5) of the overall mixed FM frequency spectra is compatible with the expected theoretical shape. Calculated main frequencies are present and the sources of noise on Ch5 (23 kHz) are visible. In mission-critical designs, this channel would be not used, reserved for low priority sensors, or assigned to low-priority and low-frequency signals. Improvement and optimization of the encoding boards such as the filtering stage should reduce this noise further, e.g., increasing the filtering order on Ch0 and Ch1 which are the most likely source of noise.

The repeatability and robustness of the signals, the low power consumption proves that tactile systems based on FM are feasible.

## 5. Conclusions

This work introduces the first example of a taxel array based on the Frequency Modulation technique. The results show the reliability and effective capability of the architecture in this use case. The transmission and reconstruction of the signal increases the amplitude of the noise from ~1% to ~2% over the 3 V sensor range which is quite small and allows for clear identification of the input signals. This working prototype presents encouraging features to be able to address scalability in artificial skin systems.

The overall current consumption of the taxel array used in this study is 36 mA at 5 V. This suggests that a 30 × 30 matrix or 900 sensors would consume 4.05 A. With the current prototype size and sensor density of 2 sensors per cm${}^{2}$, this would cover an area of 30 × 30 cm. By lowering the density, larger areas could be covered with the same power. The current encoding board consumption is currently around 1.5 mA per channel. The sensors power demand (the Hall-effect sensors used requires ~4 mA) in this and other architectures is of critical importance and needs to be as low as possible to maximize effectiveness and scalability of artificial skins. The current prototype and design version has a theoretical limit of 300 simultaneous channels per single wire, whilst maintaining the 3 kHz band spacing over the 1 MHz bandwidth. 3 kHz is the smallest frequency band possible to allow up to 100–200 Hz signal to be encoded and at the same time to retain low filter orders. A way to increase the number of sensors would be to increase the bandwidth of the IC used. Multiple subunits are envisioned to increase the number of sensors. The use of multiple subunits is a strategy to increase number of sensors accepted in literature.

The current consumption for each element is very competitive being significantly lower than two of the examples presented in the background. The flexibility to encode different sensors also makes it competitive with respect to the lower power solution, e.g., iCub skin. The reduction in wire connection number and simultaneous signal communication are important points of strength of the proposed solution in the context of scalability. A benchmark comparison between key features of the proposed architecture and existing example solutions is presented in Table 1 (Background subsection). The literature solutions use digital communication buses which require decentralized dedicated microcontrollers to digitalize and send the data. On the other hand, the proposed solution requires a more complex centralized high-speed and high-process power unit intermediary between the sensors and PC (currently CRio and FPGA or similar in the future). This unit is expected to handle the decoding of a large number of signals. The proposed solution’s ratio between sensors and decoding systems is however expected to be advantageous because the computation will grow at a significant slower pace with respect to number of sensors. This implies that the average cost per sensor will still be competitive, even if more powerful computation is required with consequent higher power demand and higher costs per single unit.

The results obtained in this research will make possible the development of larger arrays and the development of a standalone solution to enable multiple sensors to be tested and processed.

## Figures and Tables

**Table 1:** The table briefly captures key features of the Frequency Modulation (FM) approach vs. literature systems. The values are collected from papers and reviews. NOTES: (${}^{1}$) mW or mA per unit (without sensor overhead) cannot be found but the power consumption per m${}^{2}$ is competitive. (${}^{2}$) The use of microcontrollers for every 4 sensors and main bus management are assumed to have a higher power demand than the proposed solution although a specific value could not be ascertained in the published work. (${}^{3}$) Estimated from the time latency reported in the work.

-	FM Based	iCub Skin	Cut-and-Paste	Hex-o-Skin
Power consumption	6.6 mW per unit or 1.3 mA each encoding unit	5 W/m${}^{2}$ ${}^{1}$	LED 50 mA each Mitigation needed Power for uC ${}^{2}$	200 mA LED, 50 mA uC Or 47–50 mA (mitigation)/unit
Sensor methodology	Multiple types	Capacitive	LEDs	7 sensors/unit
Frequency response	Variable (e.g., up to 100 Hz or more) bands	Up to 50 Hz or 500 Hz spatial average	51 ms sampling time 1024 sensors = 20 Hz ${}^{3}$	Update rate with 8 units = 1 kHz
Processing Complexity	Simple demodulation and filtering math/Direct measurement	Direct measurement	Direct measurement	Direct measurement
Communication protocol	Single conductor (FM based)	CAN Bus 1 Mbps	SMBbus and In body-Lan 20 MHz	Local and UART 10/12 Mb/s

**Table 2:** Signal noise amplitude.

Channel	Input	Output
0	0.0223 V	0.0727 V
5	0.0237 V	0.3067 V

## Data Availability

The datasets used in this work can be found at https://doi.org/10.15125/BATH-01043 [37].
